# Diffuse Optical Spectroscopy and Imaging to Detect and Quantify Adipose Tissue Browning

**DOI:** 10.1038/srep41357

**Published:** 2017-02-01

**Authors:** U. S Dinish, Chi Lok Wong, Sandhya Sriram, Wee Kiat Ong, Ghayathri Balasundaram, Shigeki Sugii, Malini Olivo

**Affiliations:** 1 Bio Optical Imaging Group, Singapore Bioimaging Consortium, Agency for Science Technology and Research (A*STAR), Singapore; 2 Fat Metabolism and Stem Cell Group, Singapore Bioimaging Consortium, Agency for Science Technology and Research (A*STAR), Singapore; 3 Cardiovascular and Metabolic Disorders Program, Duke-NUS Medical School, Singapore; 4 School of Physics, National University of Ireland Galway, Ireland

## Abstract

Adipose (fat) tissue is a complex metabolic organ that is highly active and essential. In contrast to white adipose tissue (WAT), brown adipose tissue (BAT) is deemed metabolically beneficial because of its ability to burn calories through heat production. The conversion of WAT-resident adipocytes to “beige” or “brown-like” adipocytes has recently attracted attention. However, it typically takes a few days to analyze and confirm this browning of WAT through conventional molecular, biochemical, or histological methods. Moreover, accurate quantification of the overall browning process is not possible by any of these methods. In this context, we report the novel application of diffuse reflectance spectroscopy (DRS) and multispectral imaging (MSI) to detect and quantify the browning process in mice. We successfully demonstrated the time-dependent increase in browning of WAT, following its induction through β-adrenergic agonist injections. The results from these optical techniques were confirmed with those of standard molecular and biochemical assays, which measure gene and protein expression levels of UCP1 and PGC-1α, as well as with histological examinations. We envision that the reported optical methods can be developed into a fast, real time, cost effective and easy to implement imaging approach for quantification of the browning process in adipose tissue.

Adipose tissue has been studied extensively for its essential role in many endocrine processes and metabolic diseases such as diabetes, cardiovascular diseases and others. Adipose tissue is mainly classified into two types: white adipose tissue (WAT) and brown adipose tissue (BAT). WAT is specialised in storing energy through incorporation of triglycerides in large unilocular lipid droplets whereas BAT is specialised in burning energy as it contains abundant mitochondria, multilocular lipid droplets and generates heat. WAT is further classified mainly into two depot types: subcutaneous and visceral adipose tissue. The adipose tissue is an endocrine organ and is implicated in obesity. Obesity is caused by excessive accumulation of WAT and increases the risk of metabolic complications like diabetes^1^, cardiovascular diseases^2^,^3^,^4^,^5^ and arthritis^6^.

It was recently reported that certain WAT depots especially those classified in subcutaneous adipose tissue can be converted to a “brown-like” or “beige” state, upon exposure to stimuli such as cold or β-adrenergic agonists like CL 316,243 hydrate^7^,^8^,^9^. This process is called browning of WAT and is characterized by the appearance of scarcely populated sections of multilocular adipocytes and increased uncoupling protein 1 (UCP1)-positive adipocytes. The discovery of BAT in adult humans^10^,^11^,^12^,^13^,^14^ has sparked the interest among adipose tissue biologists and those who study exercise, metabolism and energy expenditure as to whether there is browning in humans as well. Whether browning of WAT occur in humans remains inconclusive, but recent compelling evidence shows that human BAT found in the supraclavicular region may be more beige-like type and that subcutaneous WAT has browning capability^14^,^15^.

Traditionally to assess browning of WAT, RNA and protein from WAT have to be extracted to measure gene expression levels by quantitative PCR (qPCR) and protein levels by Western blotting, respectively. These are used to examine the classical markers for browning such as UCP1 and peroxisome proliferator-activated receptor-γ coactivator (PGC)-1α. In addition, the tissues need to be paraffin embedded and sectioned for Hematoxylin and Eosin (H&E) staining to estimate the abundance of multilocular adipocytes and/or for UCP1 immunohistochemistry (IHC) to confirm browning. All of the above mentioned processes take several hours to a few days to complete in order to confirm that browning of WAT has indeed occurred. Moreover, exact quantification of the overall process is not readily available from any of the aforementioned methods.

Optical spectroscopy techniques have the unique advantage to serve as a promising tissue diagnostic tool because it provides quantitative information compared to the conventional histopathological analysis, which is more of a qualitative analysis tool. Moreover, optical techniques are relatively inexpensive, easy to use and implement. Among the various optical techniques, diffuse reflectance spectroscopy (DRS) and its complementary imaging options, multispectral imaging (MSI) and hyperspectral imaging (HSI) are highly versatile because they are dependent on the tissue optical parameters. Though the basic principle behind MSI and HSI are indeed the same, MSI is realized by imaging reflected light within several specific bands of the electromagnetic spectrum. MSI usually has three to ten different band measurements in each pixel of images produced, while HSI measures reflected light at many bands than multispectral sensors. Hyperspectral images can typically contain as many as tens of continuous spectral bands^16^. Due to the strong light absorption of hemoglobin and other tissue components in the visible near infra-red (Vis-NIR) region, DRS and MSI imaging offer great value for classification and analysis of tissue samples^17^,^18^,^19^.

DRS, MSI and HSI are well established techniques and they have been used extensively for a variety of applications in biomedicine such as in brain studies^20^,^21^,^22^,^23^, cancer detection^24^,^25^,^26^,^27^, skin research^28^,^29^,^30^, biopsy^31^,^32^, etc. However, to the best of our knowledge, there are very few reports on the application of these techniques for the detection and imaging of adipose tissues. Here, we demonstrate the application of DRS and MSI for quantitative evaluation of the browning process in adipose tissues. We used subcutaneous WAT and classical BAT from mice with or without browning stimulation to perform the study. We induced browning for either 4 days or 7 days by β-adrenergic administration to show a time-dependent increase in browning of WAT. We also carried out RNA/protein isolation, qPCR and Western blot analysis for UCP1 and PGC-1α as well as histological analysis and UCP1 immunohistochemistry to correlate with the results that we obtained using optical methods.

## Results

### Diffuse Reflectance Spectroscopy can detect and quantify adipose browning

**Figure 1:**
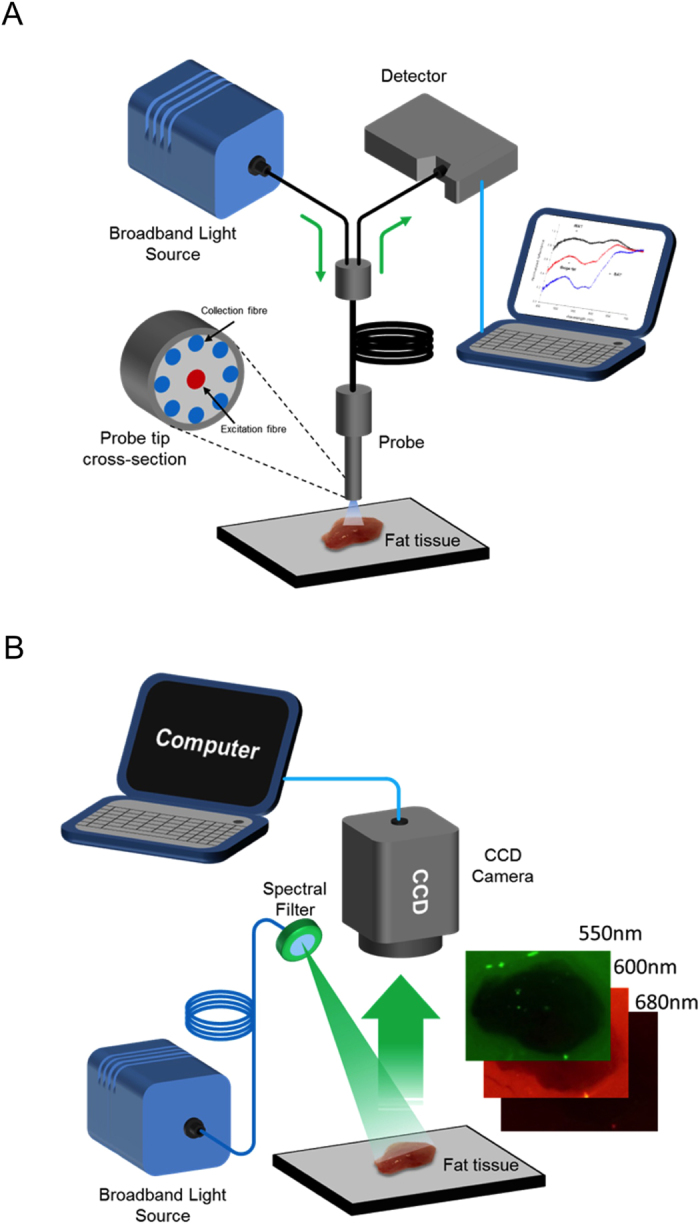
Schematic of the optical fiber based set up of DRS (**A**) and MSI (**B**). The images were drawn by D.U.S. and W.C.L., with the help of Mr. Douglas.

**Figure 2:**
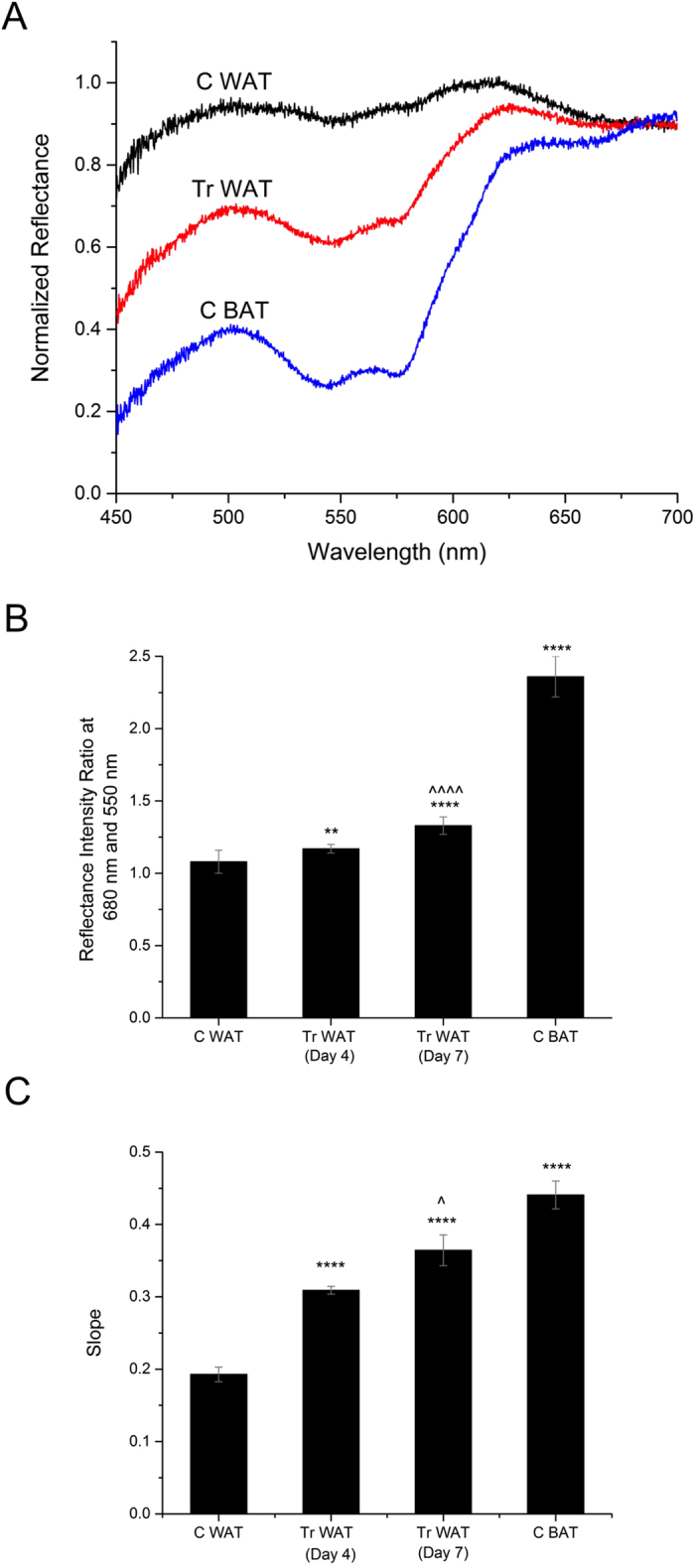
Representative normalized DRS spectra from adipose tissues (**A**). Reflectance intensity ratio at 680 and 550 nm of adipose tissues (**B**) and slope value in the in the 570–630 nm range for adipose tissues (**C**). **p < 0.01 and ****p < 0.0001 denote significance when compared to C WAT; ^p < 0.05 and ^^^^p < 0.0001 denotes significance in Tr WAT (Day 7) when compared to Tr WAT (Day 4) (n = 4–8).

Schematic of the fiber optic probe based DRS and MSI system is shown in Fig. 1. Representative reflectance spectrum of C WAT, Tr WAT and C BAT are shown in Fig. 2A. It clearly indicates that there is a marked difference in spectral profile, specifically in the 550–650 nm range, between the tissues. It is obvious that slope of the spectrum in the range around ~550–630 nm is also significantly different where the reflectance is higher for C WAT. For quantitative analysis of browning using intensity ratio, we chose two wavelengths at 680 nm and 550 nm, because at these wavelengths the spectra showed minimal and maximal differences, respectively. As shown in Fig. 2B, the mean intensity ratio value for C WAT (average of Day 4 and Day 7), Tr WAT (Day 4), Tr WAT (Day 7), and C BAT (average of Day 4 and Day 7) are 1.08, 1.17, 1.33 and 2.36, respectively. The result shows that there is an obvious increase in the value from C WAT to C BAT and the value for Tr WAT (Day 4) and Tr WAT (Day 7) are increased compared to the C WAT. This study establishes that there is about 25% increase in the intensity ratio value for Tr WAT (Day 7) when compared to C WAT. We further observed that there is no significant change in the value for C BAT and Tr BAT at Day 4 and Day 7 after CL injection (Fig. S1 in Supplementary Information (SI)).

The quantification of the slope variation in the range 570–630 nm is shown in Fig. 2C and it indicates a linear increase in the value from 0.19 to 0.44 when we compare from C WAT to C BAT. Slope value is increased from 0.19 (C WAT) to 0.30 and 0.36 for Tr WAT (Day 4) and Tr WAT (Day 7) respectively. We found that compared to the intensity ratio, slope analysis gives a clearer differentiation between Tr WAT at Day 4 and Day 7. As in the previous case, there is no difference in the slope value for C BAT and Tr BAT at Day 4 and Day 7.

### Detection and measurements of browning by Multispectral Imaging

**Figure 3:**
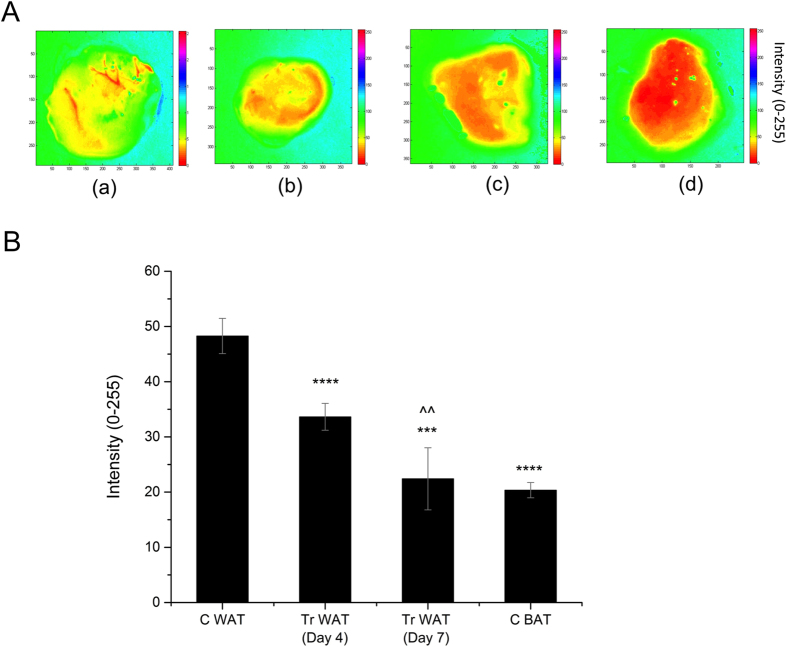
Representative MSI results of adipose tissues. (**A**) 2D Intensity map of multispectral image at 550 nm; (a) for C WAT, (b) for Tr WAT (Day 4), (c) for Tr WAT (Day 7) and (d) for C BAT (Day 7). Normalization of the image was made with respect to C WAT. Intensity values extracted for fat tissues from the MSI images at 550 nm (**B**). ***p < 0.001 and ****p < 0.0001 denotes the significance when compared to C WAT and ^^p < 0.01 denotes the significance in Tr WAT (Day 7) when compared to Tr WAT (Day 4) (n = 4–8).

MSI of the samples at various wavelengths are given in Fig. S2 (SI). We chose these wavelengths for imaging based on the significant difference in the spectra for the fat samples measured by the DRS technique. Among the various multispectral images that were acquired, we chose the images at 550 nm for estimating the browning process based on the intensity differences of the images. As shown in Fig. 3A, the observed color was lightest for C WAT, while it was darker in the case of Tr WAT (Day 4 and Day 7) and darkest for C BAT at 550 nm. This indicates the higher reflectance of the light at 550 nm from C WAT, while images of all tissues at 680 nm more or less exhibited minimal variation. The average of intensity values for these samples at 550 nm are shown in Fig. 3B. The calculated values showed a linear change from ~48 for C WAT to ~20 for C BAT. The corresponding values for Tr WAT at Day 4 and Day 7 are 33 and 23, respectively, which clearly indicates that by MSI, a simple quantification and estimation of the browning process can be achieved that complements the observed results by DRS.

### Correlation with mRNA expression and protein levels of browning markers

**Figure 4:**
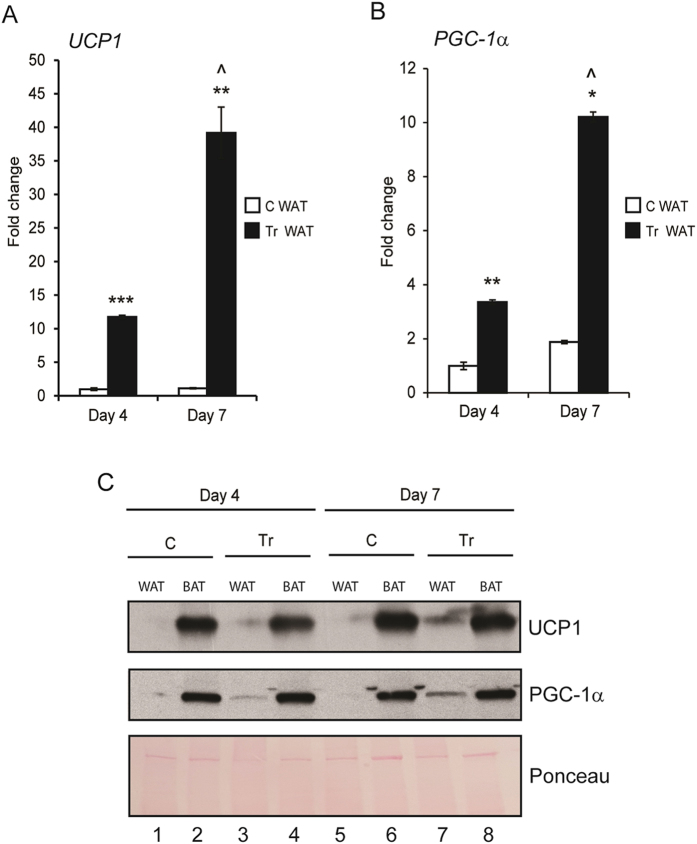
Administration of mice with CL increases UCP1 and PGC-1α mRNA expression and protein levels in WAT. mRNA expression of *UCP1* (**A**) and *PGC-1α* (**B**) in C WAT and Tr WAT at Day 4 and Day 7 post injection. *p < 0.05, **p < 0.01 and ***p < 0.001 denote significance when compared to C WAT; ^p < 0.05 denotes significance in Tr WAT (Day 7) when compared to Tr WAT (Day 4). (**C**) Western blot analysis of protein lysates obtained from Day 4 and Day 7 WAT and BAT (C/Tr) showing protein levels of UCP1 and PGC-1α. Ponceau staining was used as internal control for equal protein loading on the gel (n = 4–8).

In order to correlate our quantification of browning by DRS and MSI, we performed molecular and biochemical analyses. RNA and protein lysate were isolated from the same samples as above (C WAT, C BAT, Tr WAT, Tr BAT at Day 4 and Day 7). mRNA expression of UCP1 by qPCR analysis (Fig. 4A) revealed that CL injection induced its expression ~11.7 fold at Day 4 and ~39 fold at Day 7 in Tr WAT. As shown in Fig. 4B, *PGC-1α* expression was up-regulated ~3.3 fold at Day 4 and ~10 fold at Day 7 in Tr WAT. Figure S3 shows that BAT from both C and Tr mice have significantly higher expression of *UCP1* (A) and *PGC-1α* (B) mRNA when compared to C WAT and Tr WAT at Day 4 and Day 7. Western blot analysis also revealed that protein levels of UCP1 and PGC-1α were significantly increased in Tr WAT (Day 4), while it is further increased at Day 7 (Fig. 4C).

### H&E staining and IHC also correlate with spectroscopic measurements

**Figure 5:**
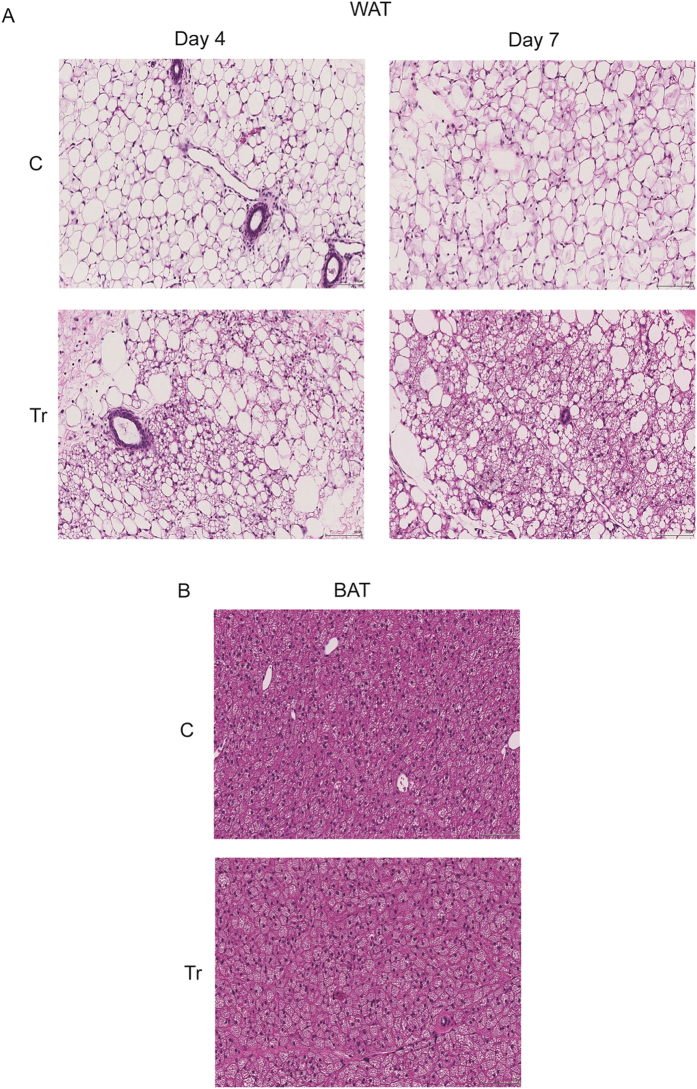
CL induces appearance of brown-like multilocular adipocytes in WAT. Representative H&E staining images (20X) in C and Tr WAT at Day 4 and Day 7 (**A**) and C and Tr BAT at Day 7 (**B**). Scale bar represents 100 μm (n = 4–8).

Histological analyses were also performed to confirm the measurement by DRS and MSI. H&E staining of Tr WAT from Day 4 revealed increased multilocular adipocytes compared to C WAT, while Day 7 Tr WAT sections showed further increased number of multilocular adipocytes (Fig. 5A), indicating that CL injection indeed induces browning in WAT in a time-dependent manner. Figure 5B depicts the dominance of multilocular adipocytes in C BAT (Day 7) and further shows that CL injection does not alter the abundance of these adipocytes in Tr BAT at Day 7.

**Figure 6:**
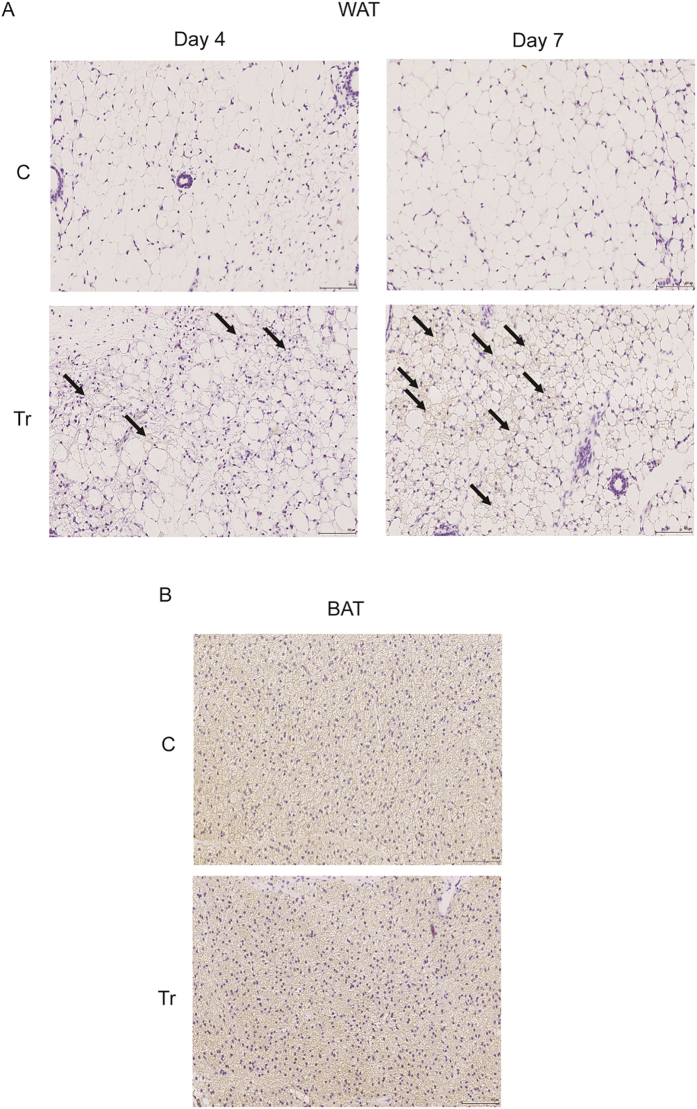
CL induces UCP1 expression in WAT, as shown by IHC. UCP1 IHC images (20X) in C and Tr WAT at Day 4 and Day 7 (**A**) and C and Tr BAT at Day 7 (**B**). The arrows indicate increased UCP1 staining in Day 4 and Day 7 Tr WAT. Scale bar represents 100 μm (n = 4–8).

Images from UCP1 IHC also correlated with the H&E staining; Day 4 Tr WAT sections revealed significantly increased levels of UCP1 staining (as indicated by arrows in Fig. 6A) compared to C WAT, while Day 7 Tr WAT showed further increase in adipocytes containing UCP1 signals (Fig. 6A), confirming that CL injection induces browning of WAT in the time-dependent manner. Figure 6B shows the high saturating levels of UCP1 that is present in nearly all adipocytes in both Day 7 C BAT and Tr BAT.

## Discussion

Over the past decade, DRS and MSI have emerged as promising clinically viable tools for disease diagnosis and monitoring^18^,^19^,^20^,^21^,^22^,^23^,^24^,^25^,^26^,^27^,^28^,^29^,^30^,^31^,^32^. In DRS, tissue characterization can be realized after illuminating it with light in a specific spectral band and detecting the diffuse reflected light. In MSI, at a given time, the sample is illuminated at specific discrete wavelength and the resulting images are captured using a camera to form images across these wavelengths. In both of these techniques, detected light is highly dependent on the tissue absorption and scattering properties. Hence, the analysis of the spectral signature provides highly specific and quantitative biochemical, morphological and functional information. In this context, it is of great importance to use these techniques for the study of adipose tissue browning.

Browning of WAT and its quantification is highly significant in understanding the mechanism of obesity and diabetes. Until now, common imaging approaches such as magnetic resonance imaging (MRI) and positron emission tomography (PET) have been used to detect classical BAT and WAT *in vivo*^33^,^34^,^35^,^36^,^37^. However, it has been a great challenge to detect the browning process of WAT both *in vivo* and *ex vivo* using these techniques. This is partly because only a certain subpopulation of white adipocytes in WAT can be induced to undergo browning, leading to partial levels of UCP1-mediated heat generation compared to classical BAT^7^,^8^,^9^. Further, existing imaging methods may not provide sufficient resolution or sensitivity to detect this population. Most common methods to detect the browning process *ex vivo* are based on molecular and biochemical analyses, which are relatively laborious, inconvenient and slow processes. Hence, our current study addresses an easy to use, time sensitive imaging method to detect the browning of adipose tissue.

Generally, WAT cells are composed of a single large intracellular lipid droplet, while BAT cells are characterized by multiple smaller intracellular lipid droplets and abundance of iron-rich mitochondria. Due to the occurrence of multilocular fat in BAT and increased number of mitochondria, extensive cytoplasm and vascular supply, its fat fraction is significantly less than that of WAT. It was demonstrated through MRI studies that the fat fraction is significantly different in BAT when compared to that of WAT^35^,^36^,^38^,^39^. This difference in fat and water content in WAT and BAT may be contributing to the spectral profile measured using DRS because both lipids and water have differential optical absorption properties in the visible-NIR range. Moreover, due to the difference in vascular supply between BAT and WAT, the observed spectral difference can also be attributed to the hemoglobin, which has characteristic optical absorption in the measured spectral window. Additionally, it was shown that expression of cytochrome c oxidase is significantly higher in browning adipose tissue upon CL injection^40^. This increase in cytochrome proteins may also lead to differences in the absorption spectrum, as described earlier^41^,^42^.

The gradual increase in the intensity ratio and slope value we obtained from control to Day 4 and Day 7 of Tr WAT clearly indicates the advancing of browning in the WAT. Our approach of using DRS and MSI for the quantification of browning process is novel because it offers a fast, real time, inexpensive and easy to implement approach. Based on our study, we could detect the browning process as early as 4 days after the injection of the β-adrenergic compound, CL 316,243 hydrate. The spectral changes observed in the tissue due to browning is quantifiable based on the intensity ratio and slope analysis in the DRS method. The MSI analysis complemented and established the validity of the DRS technique.

Quantification by DRS and MSI in general correlated well with the molecular, biochemical and histological methods, in which CL induced browning genes/proteins and appearance of brown-like adipocytes in a time-dependent manner. Though the absolute value and fold change in browning measured using the optical techniques cannot be compared directly to that of the biochemical or histological methods, this approach is unique and simple in providing a clear trend for quantification of browning of WAT.

NIR spectroscopy and DRS have been extensively used for *in vivo* detection up to a few centimetres in tissues^43^,^44^. As a general principle, the mean light penetration depth in the reflection geometry is in the order of the half the distance between source and detector fibers^43^. Hence, by optimizing the source (excitation) and detector (collection) fiber separation, interrogation of tissues at various depths can be achieved. Very recently, Ganesan *et al*. used diffuse optical technique to detect the changes in subcutaneous adipose tissue during weight loss in humans. They used the concentrations of hemoglobin, water and lipid and the wavelength-dependent light scattering amplitude and its slope for the characterization^45^. They could detect the fat *in vivo* using a probe with optimized source-detector separation. In another study, Nirengi *et al*. used NIR-time resolved spectroscopy to detect the density of BAT induced by treatment with thermogenic capsaicin analogue^46^. However, none of these studies were employed to tackle the challenging problem of detecting and quantifying the browning process of WAT. Based on our results and other reported studies on the detection of BAT, we envision that diffuse reflectance fiber probe could be developed as a simple optical device for studying the fat tissues and their cellular changes such as the browning process *in vivo*.

## Materials and Methods

### Animals

Young 6-week old balb/c nude mice were obtained from InVivos, Singapore. The mice were housed at the animal facility at Biological Resource Centre, Singapore. All animals had free access to chow diet and water. All experimental procedures were approved by the Institutional Animal Care and Use Committee (IACUC) of Biological Resource Centre, Singapore, and performed in accordance with its relevant guidelines and regulations.

### Induction of browning *in vivo*

Mice (n = 4 to 8) were injected with CL 316,243 hydrate (CL) (Sigma), 1 mg/kg body weight intra-peritoneally (I.P.). The control (C) mice were injected with equal volume of saline. CL 316,243 hydrate was injected (treatment group, ‘Tr’) daily for 4 days or 7 days. On Day 4 and Day 7, the mice were euthanized and WAT from the inguinal region (C WAT, Tr WAT) and BAT (C BAT, Tr BAT) from the interscapular region were isolated. The fresh tissues were immediately used for DRS and MSI, snap frozen for RNA and protein expression analyses, and/or stored in 10% normal buffered formalin (NBF) for paraffin embedding.

### DRS set up and spectral acquisition

Fiber optic probe based DRS setup consists of a halogen light source (Ocean optics HL-2000-FHSA) connected to a VIS-NIR reflectance source/detector fiber optic probe (Ocean optics QR600-7-VIS-125F) with 7 fibers each with 600 μm core fibers using a SMA connector. Fiber consists of a central excitation fiber and a ring of collection fibers. Fiber probe was placed perpendicular to the sample surface. Detected signal was fed into a spectrometer (Ocean optics USB 4000) and to a computer for data processing. Integration time was set at 100 ms throughout the spectral acquisition. Initially, dark spectrum was measured by keeping the lamp off and then reference spectrum is obtained using a reflectance standard. After subtracting the dark spectrum, the reflectance from fat samples was measured by placing it on a translational stage and distance of the fiber probe from the sample surface was kept constant throughout the experiment. Beam spot on the tissue sample was maintained at about 4 mm. Diffuse reflectance spectra were measured in the range 450–700 nm. Spectral measurements were done at least 5 locations on the tissue samples and throughout the experiment, excitation light intensity was kept constant.

Quantification of the browning process of the fat tissues was evaluated using two approaches, namely intensity ratio and slope analysis. Intensity ratio was calculated at two wavelengths- 680 nm and 550 nm, while the slope of the curve was determined in the wavelength range 570–630 nm. Normalization processes were carried out using the slope value of C WAT.

### MSI setup and image analysis

In MSI, light excitation was provided by the same halogen source that was used for the DRS measurement. In order to excite the sample at a given wavelength, band pass filters (Thorlabs) were used and imaging was carried out at 550, 600 and 680 nm. Images were acquired using a camera (Nikon D 7000) fitted with a lens (AF-S NIKKOR 18–105, 3.5–5.6) followed by image processing. Acquisition time of the camera was set at 4 seconds. Diameter of the light beam was adjusted such that it uniformly illuminates the whole tissue sample surface. As shown in Fig. 1B, the excitation fiber probe and camera position were fixed in all imaging measurements using a translational stage with a holder. Incident angle (30°) of the light beam was also fixed throughout the experiment to achieve reproducible data. Then, a home-built Matlab program was used for image intensity extraction. The average intensity value from all of the pixels inside a fixed region in the image was calculated. Normalization of the image was made with respect to C WAT.

### Real-time qPCR

Total RNA was extracted using the RNeasy Lipid Tissue Mini Kit (Qiagen) according to the manufacturer’s instruction, following mechanical disruption of the adipose tissues. cDNA conversion was performed using the RevertAid H minus first strand cDNA synthesis kit (Fermentas). qPCR was performed using SYBR Green PCR Master Mix on a StepOnePlus Real-Time PCR System (Applied Biosystems) using the primer pairs: *UCP1*_F: 5′-GGCCTCTACGACTCAGTCCA-3′; *UCP1*_R: 5′- TAAGCCGGCTGAGATCTTGT-3′; *PGC-1α*_F: 5′-AGCCGTGACCACTGACAACGAG-3′; *PGC-1α*_R: 5′-GCTGCATGGTTCTGAGTGCTAAG-3′; *β-ACTIN*_F: 5′-ACCTTCTACAATGAGCTGCG-3′; *β-ACTIN*_R: 5′-AGGTCTTTACGGATGTCAACG-3′. Relative mRNA were calculated and normalized to the level of *β-ACTIN*.

### Western Blot analysis

Total protein lysates from adipose tissues were obtained by mechanical disruption in RIPA lysis buffer. The protein concentrations of lysates were determined using Bradford’s assay. 20 μg of protein was separated on 4–20% Mini-Protean gels (Biorad) transferred to nitrocellulose membrane by electro blotting. The membranes were then probed with primary antibodies, UCP1 (Abcam; 1:1000 dilution) and PGC-1α (Abcam; 1:1000 dilution). Ponceau stain was used to determine equal protein loading on the gel. Following subsequent washes and incubation with respective secondary antibodies, the horseradish peroxidase activity was detected using chemiluminescent reagents.

### Hematoxylin and Eosin (H&E) staining and Immunohistochemistry (IHC)

Following the collection of the adipose tissues and storing in 10% NBF, they were embedded in paraffin and sectioned. H&E staining and UCP1 IHC were performed by the Advanced Molecular Pathology Laboratory (AMPL) at the Institute of Molecular and Cell Biology (IMCB), A*STAR, according to standard operating procedures. 1:100 dilution of UCP1 antibody (Abcam) was used for UCP1 IHC.

### Statistical analysis

The p value was calculated using ANOVA for multiple comparisons with corrections and p < 0.05 being considered as significant. Four to eight mice were used for the experiments. The results are presented as mean ± SE.

## Additional Information

**How to cite this article**: Dinish, U. S. *et al*. Diffuse Optical Spectroscopy and Imaging to Detect and Quantify Adipose Tissue Browning. *Sci. Rep.* **7**, 41357; doi: 10.1038/srep41357 (2017).

**Publisher's note:** Springer Nature remains neutral with regard to jurisdictional claims in published maps and institutional affiliations.

## Supplementary Material

Supplementary Figures
