# Supplementary material for: Diffuse Optical Spectroscopy and Imaging to Detect and Quantify Adipose Tissue Browning
Source: Sci Rep. 2017 Feb 1;7:41357. doi: 10.1038/srep41357 (PMC5286412; doi:10.1038/srep41357)
Supplement: Supplementary Figures [file srep41357-s1.pdf]

Supplementary Information

Diffuse Optical Spectroscopy and Imaging to Detect and Quantify Adipose Tissue Browning

U. S Dinish[1#], Chi Lok Wong[1#], Sandhya Sriram[2#], Wee Kiat Ong[2], Ghayathri Balasundaram[1], Shigeki Sugii[2,3*], Malini Olivo[1,4*]

[1]Bio Optical Imaging Group, Singapore Bioimaging Consortium, Agency for Science Technology and Research (A*STAR), Singapore

[2]Fat Metabolism and Stem Cell Group, Singapore Bioimaging Consortium, Agency for Science Technology and Research (A*STAR), Singapore

[3]Cardiovascular and Metabolic Disorders Program, Duke-NUS Graduate Medical School, Singapore

[4]School of Physics, National University of Ireland Galway, Ireland

# Authors contributed equally

*Corresponding authors: shigeki_sugii@sbic.a-star.edu.sg

: malini_olivo@sbic.a-star.edu.sg

FIGURE LEGENDS

Figure S1. Reflectance intensity ratio of adipose tissues at 680 nm and 550 nm. ####p<0.0001 denotes the significance when compared to Tr WAT.

Figure S2. MSI of the adipose tissues at 550 nm, 600 nm and 680 nm. Average intensity is displayed in a scale of 0-255.

Figure S3. mRNA expression of *UCP1* (A) and *PGC-1α* (B) in C, Tr WAT and BAT at Day 4 and Day 7. *p<0.05, **p<0.01, ***p<0.001 and ****p<0.0001 denote significance when compared to C WAT; ^p<0.05 denotes significance in Tr WAT (Day 7) when compared to Tr WAT (day 4); ##p<0.01, ###p<0.001, ####p<0.0001 denote significance in Tr BAT when compared to Tr WAT.

Fig. S1

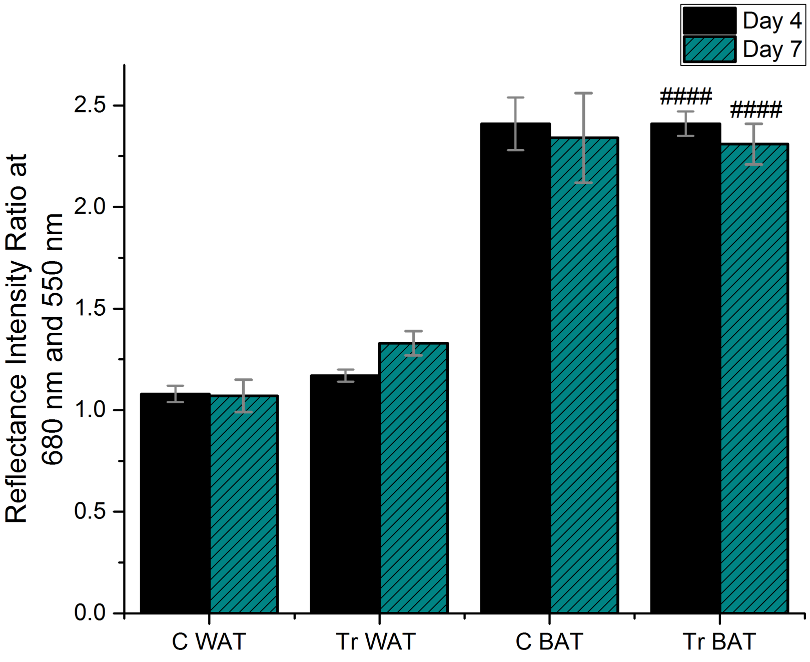
Day 4
Day 7
Reflectance Intensity Ratio at 680 nm and 550 nm
2.5
2.0
1.5
1.0
0.5
0.0
####
####
C WAT
Tr WAT
C BAT
Tr BAT

Fig. S2

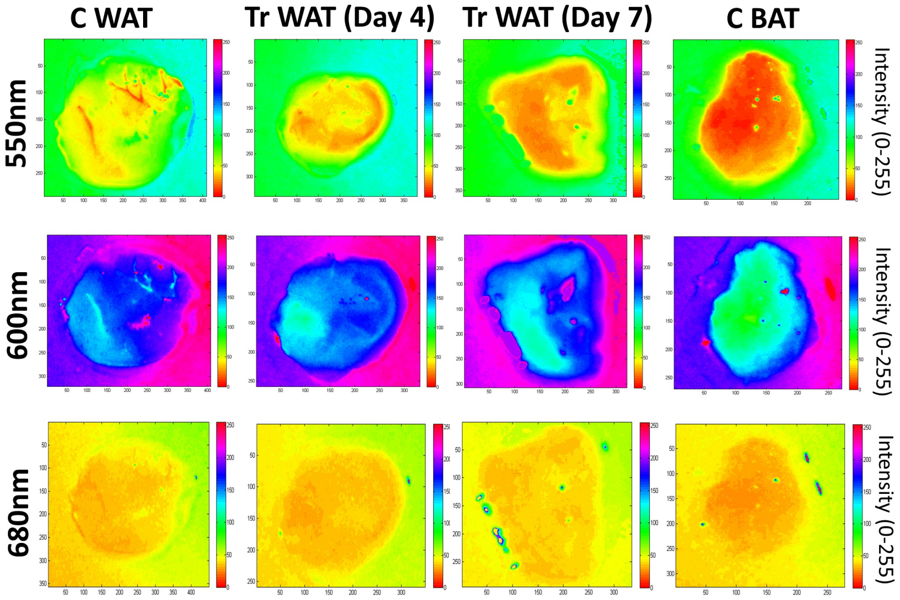
C WAT
Tr WAT (Day 4)
Tr WAT (Day 7)
C BAT
550nm
600nm
680nm
Intensity (0-255)
Intensity (0-255)
Intensity (0-255)

Fig. S3

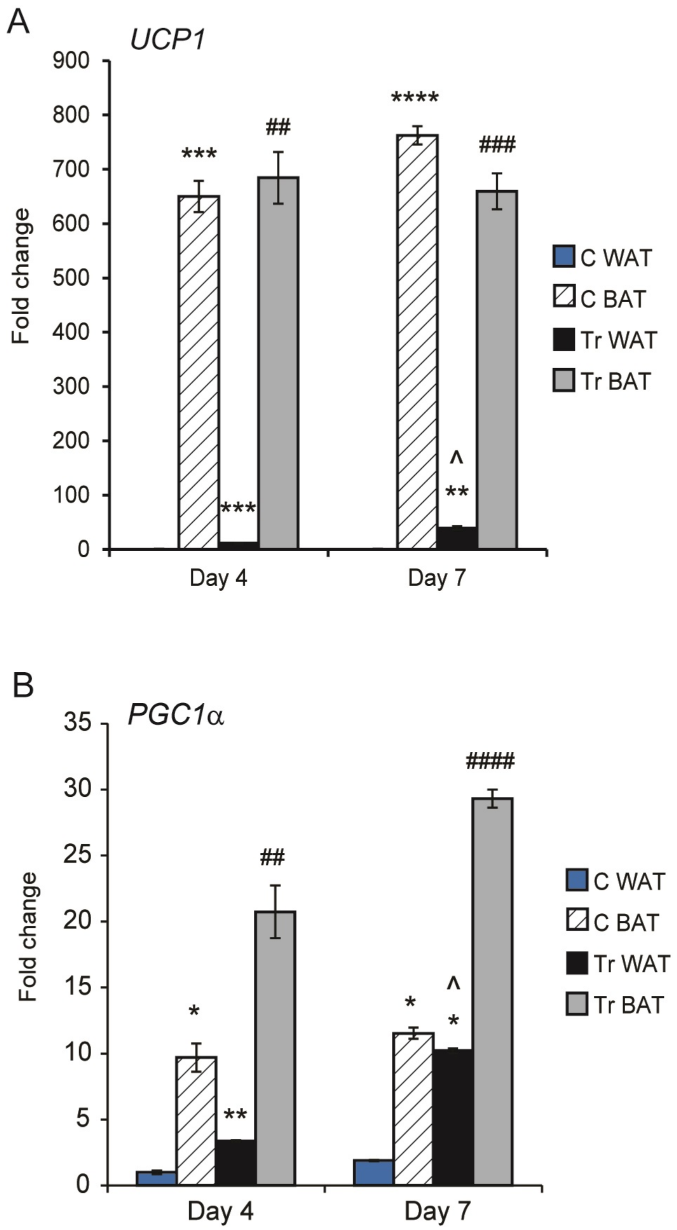
A
UCP1
Fold change
900
800
700
600
500
400
300
200
100
0
***
##
****
###
***
^
**
Day 4
Day 7
C WAT
C BAT
Tr WAT
Tr BAT
B
PGC1α
Fold change
35
30
25
20
15
10
5
0
####
##
*
**
*
^
*
Day 4
Day 7
C WAT
C BAT
Tr WAT
Tr BAT
